# Total and Proteinase K-Resistant α-Synuclein Levels in Erythrocytes, Determined by their Ability to Bind Phospholipids, Associate with Parkinson’s Disease

**DOI:** 10.1038/srep11120

**Published:** 2015-06-11

**Authors:** Suaad Abd-Elhadi, Asaf Honig, Dganit Simhi-Haham, Meir Schechter, Eduard Linetsky, Tamir Ben-Hur, Ronit Sharon

**Affiliations:** 1 Biochemistry and Molecular Biology, IMRIC, The Hebrew University-Hadassah Medical School; 2 Department of Neurology, The Agnes Ginges Center for Human Neurogenetics, Hadassah - Hebrew University medical Center.

## Abstract

A marker for diagnosis of Parkinson’s disease (PD), which reflects on the occurrence of peripheral pathogenic mechanisms, would potentially improve therapy. The significance of α-Synuclein (**α-**Syn) expression in red blood cells (RBC) is currently unclear. Here we investigated whether RBC’s-expressed **α-**Syn may associate with PD. To this aim, we determined the levels of total and proteinase K-resistant α-Syn in samples of packed red blood cells (PRBCs). Twenty-one individuals with PD at various disease stages and 15 healthy controls, with similar demographic features, were recruited to this study. **α-**Syn levels were determined by their biochemical property to bind phospholipids, using a phospholipid-ELISA assay. A significantly lower ratio of total-to-proteinase K-resistant **α-**Syn levels was detected in PD patients than in the healthy control group. However, there was considerable overlap between the two groups. Suggesting a need for additional markers to be tested in combination with **α-**Syn levels. To the best of our knowledge, this is the first evidence for an association between RBCs-expressed **α-**Syn and pathogenic mechanisms involved in PD.

Parkinson’s disease (PD) is a heterogenic disease with complex clinical, pathological and genetic features (reviewed in^1^). α-Synuclein (α-Syn) protein is implicated in the genetics and pathology of PD (reviewed in^2^). In the brain, α-Syn pathology, in the form of Lewy pathology, is strongly associated with the progression of the disease at the histopathology and symptomatic levels^3^,^4^.

α-Syn pathology is also detected in the peripheral autonomic nervous system of PD patients, specifically, within the gastrointestinal system, were it may precede the CNS pathology (for a recent reviews, see^5^,^6^). The occurrence of α-Syn pathology in the peripheral nervous system before the onset of PD may support the Braak hypothesis^4^, which proposes that α-Syn pathology spreads in a predictable and sequential manner, affecting the dorsal motor nucleus of the Vagus nerve at an early stage of the disease and only involving the substantia nigra pars compacta (SN_C_) in a later stage of the disease. Recent studies provide evidence supporting the spread of α-Syn from the peripheral to the central nervous system^7^,^8^,^9^.

α-Syn expression was recently detected in blood cells, primarily in megakaryocytic and erythroid lineages^10^,^11^,^12^,^13^, with high levels of expression detected in red blood cells (RBCs)^10^ and platelets^11^,^14^. α-Syn was found to share regulatory mechanisms with the expression of heme metabolism proteins. GATA transcription factors were shown to regulate the expression of α-Syn in the blood^15^. These factors are well known for their role in blood cell development and differentiation. Hematologic abnormalities were demonstrated in α-Syn null mice^16^. Suggesting a role for α-Syn in the normal development/ differentiation and function of blood cells.

It is currently not clear whether α-Syn expression in blood cells may associate with the pathogenic mechanisms involved in PD. Unlike post-mitotic neurons in aged PD brains, the pool of RBCs, which express the highest levels of α-Syn^10^, is continuously renewed with a relatively short life spun of 120 days. This feature of RBCs is not expected to allow for deposition of aggregated proteins or inclusion formations, which are the hallmark of α-Syn pathology in PD and the synucleinopathies^4^. Yet, specific post-translationally modified forms of α-Syn in blood cells may potentially correlate with pathogenic mechanisms and with PD. In this regard, a higher degree of nitrotyrosine-modified α-Syn was reported in peripheral blood mononuclear cells (PBMCs) from PD patients^17^.

In contrast to blood cells-expressed α**-**Syn, the extracellular α**-**Syn has attracted considerable interest in the past decade. These extracellular forms of α**-**Syn, detected in accessible body fluids like blood-plasma and cerebrospinal fluid (CSF), are suspected to play a role in a prion-like cell-to-cell transfer of α**-**Syn pathology and reflect on the pathogenic process in the brain. Indeed, it was suggested that α-Syn detected in the CSF is principally derived from neurons of the central nervous system (CNS)^18^.

In this study we focused on **α-**Syn derived from packed red blood cells (PRBCs) and asked whether RBC’s **α-**Syn may associate with Parkinson’s disease. Total and proteinase K-resistant **α-**Syn levels were determined by a quantitative and sensitive phospholipid-ELISA method that we have recently developed^19^. A significantly lower ratio of total-to-proteinase K-resistant **α-**Syn levels was detected in PD patients than in the healthy control group. However, there was considerable overlap between the two groups. Our results suggest that **α-**Syn derived from PRBC may potentially be used as an indicator for PD.

## Results

### The detection of α-Syn in packed red blood cells (PRBCs) is interrupted by the presence of peroxidases and phospholipid-binding proteins

**Figure 1:**
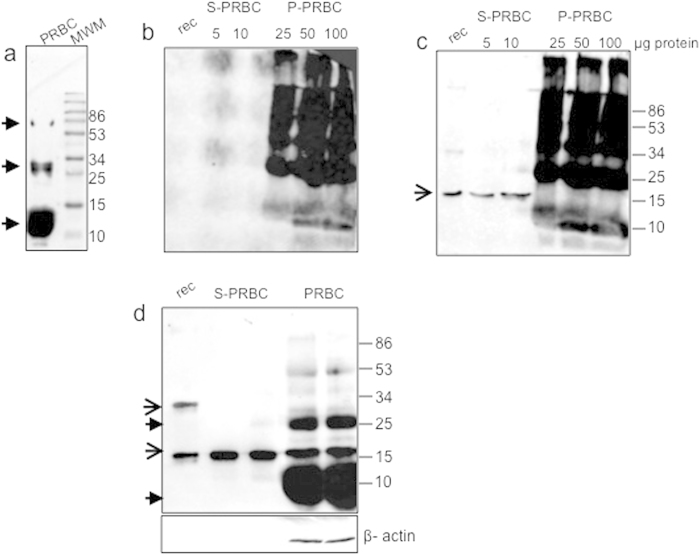
Detection of α-Syn in samples of packed red blood cells (PRBC). (**a**) A PRBCs sample (10 μg proteins), separated on a 10%SDS-PAGE, transferred to nitrocellulose membrane and reacted directly with a chemiluminescence (ECL) detection kit (Pierce, CT, USA), without prior incubation in primary or secondary antibodies. (**b**) Samples of PRBC were heated at 100 °C for 10 minutes, followed by a spin down at 20,000 × g, to generate the soluble (S)-PRBC and pellet (P)-PRBC. Protein samples (at the indicated amounts) were separated on a 10%SDS-PAGE and the blot was reacted directly with an ECL detection kit as in (**a**). (**c**) The blot in (**b**) reacted with anti **α-**Syn antibody (Syn #10^19^) and a secondary donkey anti mouse-HRP conjugated antibody. Arrow, an **α-**Syn-specific signal. (**d**) Protein samples of PRBC or S-PRBC, analyzed by Western blotting as in (**c**). Filled arrow, non specific bands; line arrow, **α-**Syn-specific bands.

To find out whether phospholipid binding may support a quantitative and sensitive capture of α-Syn from PRBCs, we tested the efficacy of the phospholipid ELISA method to capture α-Syn from samples of osmosis-lysed PRBCs. For this aim, we attempted to determine the amount of α-Syn as a function of sample volume. The results suggested that α-Syn’s capture from PRBCs by phospholipid ELISA is disturbed by the presence of additional phospholipid-binding proteins as well as peroxidases enriched in RBCs. We found that peroxidases and catalase in RBCs, directly react with the peroxide reagent present in the detection step of the phospholipid-ELISA and the chemiluminescence (ECL) detection kit in Western blotting. In control experiment, in which both the primary and secondary antibodies were omitted, strong ECL-responsive signals were observed in samples of PRBC (Fig. 1a). To eliminate the effect of these undesired factors, all PRBC samples were pre-treated at 100 °C for 10 minutes, followed by a spin down at 20,000 × g, to remove the denatured proteins. This procedure resulted in a dramatic (~90%) reduction in protein concentration of the soluble, 20,000 × g fraction (S-PRBC) and the formation of a 20,000 × g pellet fraction (P-PRBC). Importantly, following this heat treatment, direct ECL-responsive signals were observed in P-PRBC but not in S-PRBC samples, when the primary and secondary antibodies were omitted (Fig. 1b).

Next, the occurrence of **α-**Syn was tested by Western blot in samples containing 5 and 10 μg of protein from S-PRBCs and 25–100 μg from P-PRBCs, using the anti-human α-Syn antibody, Syn #10^19^. The blot showed a specific α-Syn immunoreactive band migrating as a 17 kDa monomer in the S-PRBC fraction; this band was not detected in the P-PRBC fraction (Fig. 1c). Equal aliquots of lysed PRBCs (10 μg protein) were either pre-treated at 100 °C and spun down to generate S-PRBC or left untreated prior to analysis by Western blotting with anti-α-Syn and anti- β-actin antibodies. The result (Fig. 1d) suggests the occurrence of highly similar α-Syn levels in samples of untreated PRBCs and treated S-PRBCs. β-actin signal, detected in PRBC samples, was not detected in S-PRBC samples, suggesting that following the heat treatment, β-actin was absent from the soluble fraction. Together, these results suggest that the 100 °C treatment enabled a partial purification of α-Syn from lysed PRBCs.

### Phospholipid ELISA for the detection of α-Syn in packed red blood cells

**Figure 2:**
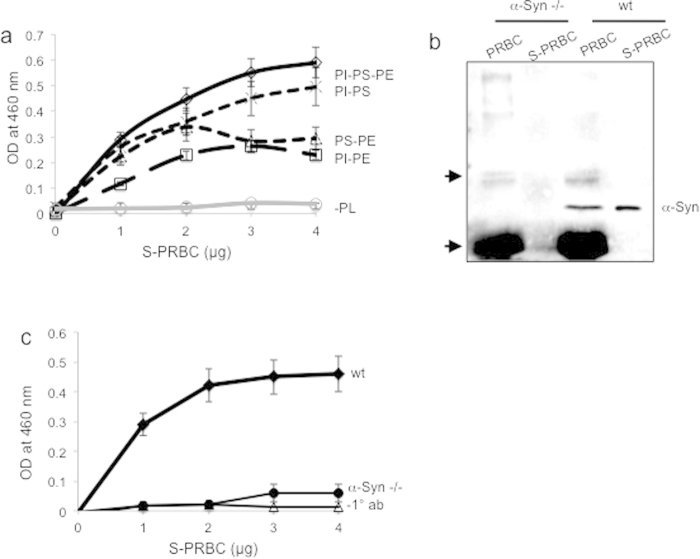
The phospholipid ELISA assay specifically detects α-Syn in S-PRBC samples. (**a**) Samples of human S-PRBC (0–4 μg protein) were applied to a 96-well microtiter dish containing the indicated phospholipids (Phosphatidylinositol (PI); phosphatidylserine (PS); phosphatidylethanolamine (PE)) at a final amount of 100 μg/well, or without phospholipids (-PL) and processed for the detection of α-Syn using anti human α-Syn antibody, α-Syn #10^19^). Graph illustrates the means ± SD, n = 3 replicates. (**b**) Samples of mouse PRBC or S-PRBC from wt or α-Syn-/- (1 μg protein), analyzed by Western blotting using anti- α-Syn antibody, α-Syn #3. Arrows indicate non-specific immunoreactive bands. (**c**) S-PRBC from wt and α-Syn-/- mice were applied in increasing amounts (0–4 μg protein) into wells of a microtiter dish coated with PI-PS-PE phospholipids (as in (**a**)), using anti α-Syn antibody, α-Syn#3 or without a primary detecting ab (−1**°** ab). Graph illustrates the means ± SD of n = 3 repeats.

We sought to find out which phospholipids maximize α-Syn detection in human PRBCs. For this aim, samples of S-PRBC were tested by phospholipid ELISA. A graph representing the capture of α-Syn as a function of sample size (0-4 μg protein) was obtained for PI-PS-PE; PI-PS; PS-PE and PI-PE (in declining order; Fig. 2a). The calculated correlation coefficient for the capture of α-Syn by PI-PS-PE was R^2^ = 0.94. The detection of α-Syn by binding to phospholipids was specific and depended on the presence of phospholipids in the microtiter wells (Fig. 2a).

To determine the specificity of the phospholipid-ELISA to detect **α-**Syn in S-PRBC samples, we examined **α-**Syn in samples of mouse S-PRBC, obtained from either wild type (wt) or α-Syn-/- mice by Western blotting (Fig. 2b) and in parallel, by phospholipid ELISA with PI-PS-PE (Fig. 2c). For the detection of mouse **α-**Syn we used an anti **α-**Syn ab that recognizes both human and mouse **α-**Syn. An anti **α-**Syn antibody-dependent signal was observed in S-PRBC from wt mice but not from **α-**Syn-/- mice by both methods. Together, the results suggest that the phospholipid-ELISA assay specifically detect **α-**Syn protein in samples of RBCs.

**Figure 3:**
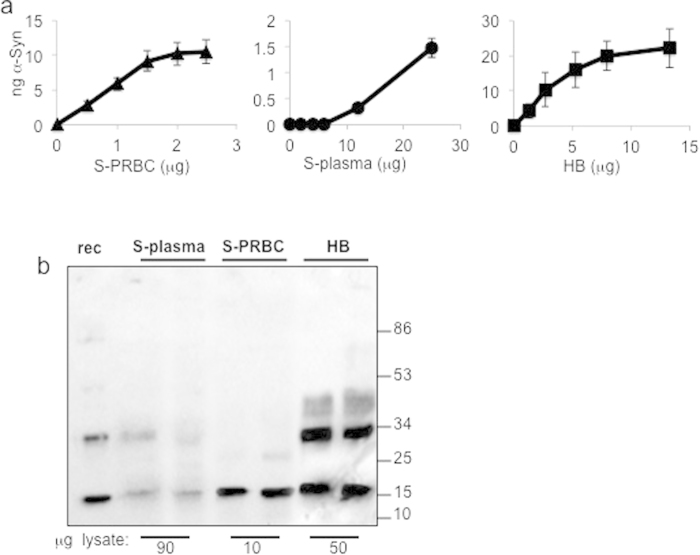
α-Syn levels in S-PRBC, S-plasma and soluble fraction of human brains (**a**) Samples of S-PRBC (0–2.5 μg protein); S-plasma (0–25 μg protein) or the soluble fraction of human brain (HB, 0–13 μg protein) were analyzed by phospholipid-ELISA using PI-PS-PE for capture and anti **α-**Syn ab, **α-**Syn#10^19^, for detection. (**b**) Samples of S-plasma, S-PRBC and HB (at the indicated protein amounts), analyzed by Western blotting using anti **α-**Syn ab, **α-**Syn#10. A representative blot out of n = 3.

The levels of α-Syn detected by the phospholipid-ELISA method were compared between S-PRBC, S-plasma and the soluble fraction of human brains (see Methods, performed in parallel). α-Syn levels were determined according to a standard curve with known amounts of purified-recombinant α-Syn (0.006–1.2 ng/μl) using PI-PS-PE for capture. The calculated **α-**Syn levels (in ng) per μg protein in the sample were 5.96 ± 1.05 in S-PRBC; 0.035 ± 0.0021 in plasma; and 3.96 ± 0.41 α-Syn in the soluble fraction of human brain (mean ± SD of n = 4; Fig. 3a). In parallel, **α-**Syn levels were analyzed by Western blotting, probed with an anti-**α-**Syn antibody, **α-**Syn #10 (Fig. 3b). Quantifying the **α-**Syn signal obtained by the Western blot confirmed the results obtained by phospholipid ELISA: **α-**Syn levels detected in S-PRBC were ~1.5–2 fold higher than the levels detected in the soluble fraction of human brain and ~160 fold higher than the levels detected in the plasma.

### Proteinase K-resistant α-Syn occurs in samples of PRBCs

**Figure 4:**
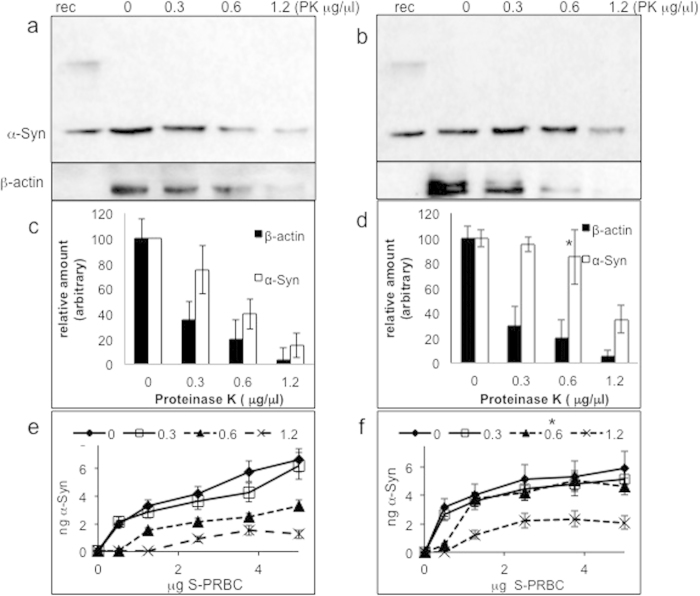
Higher levels of proteinase K-resistant α-Syn in PD samples. Western blot analysis of PRBC samples (20 μg protein) of healthy controls (**a**) and PD patients (**b**), treated with the indicated Proteinase K concentrations for 30 minutes at 37 °C and spun at 20,000 g before loading on a 10% SDS-PAGE. A representative blot, reacted with anti **α-**Syn (**α-**Syn#10)^19^ and anti β-actin abs. Densitometry analysis of Western blots for HC (**c**) and PD (**d**). The levels of **α-**Syn and β-actin are calculated as percent of the signal obtained without proteinase K (set at 100%). Mean ± SD of n = 6 different samples; *, p < 0.01, t-test. Samples of PRBC from healthy (**e**) and PD (**f**), analyzed by Phospholipid-ELISA as in Fig. 2a. Graph present mean ± SD of n = 6.

Samples of PRBCs from healthy subjects and individuals with PD were tested for the presence of proteinase K-resistant **α-**Syn. The effect of proteinase K digestion was initially tested in a small number of samples (n = 6) and exemplified by the degradation of β-actin, which was analyzed by Western blotting and quantified in parallel, in the same samples. The results show a dramatic loss in β-actin signal, with only traces of β-actin left following incubation with 1.2 μg/μl proteinase K (Fig. 4a,b). A gradual loss in **α-**Syn signal, as a function of increasing proteinase K concentration, was observed. Specifically, for each S-PRBC sample, the **α-**Syn signal obtained without proteinase K was set at 100% and the relative **α-**Syn signal obtained with proteinase K was calculated. Higher levels of proteinase K-resistant **α-**Syn signal were obtained for the PD than for the control group, with a significantly higher level at 0.6 μg/μl proteinase K (control: 39.9 ± 3.5; PD: 78.7 ± 10.5; mean ± SD; t-test, *, p < 0.01; Fig. 4c,d).

### Proteinase K-resistant α-Syn is detectable by phospholipid ELISA

We next asked whether proteinase K-resistant α-Syn is detectable by phospholipid binding. Proteinase K-treated and untreated samples were subjected to detection of **α-**Syn by phospholipid-ELISA, using PI-PS-PE for capture of **α-**Syn. The results suggest a correlation between the signals for proteinase K-resistant **α-**Syn obtained on the Western blot (Fig. 4a–d) and using phospholipid ELISA (Fig. 4e,f): in samples treated with 0.6 μg/μl proteinase K, proteinase K-resistant **α-**Syn levels were significantly higher for PD subjects (1.77 ± 0.86) than for healthy controls (0.70 ± 0.45) Mean ± SD; p < 0.01, t-test. This concentration of proteinase K was used for further analyses.

### α-Syn resistance to proteinase K digestion is dependent on its associations with phospholipids

**Figure 5:**
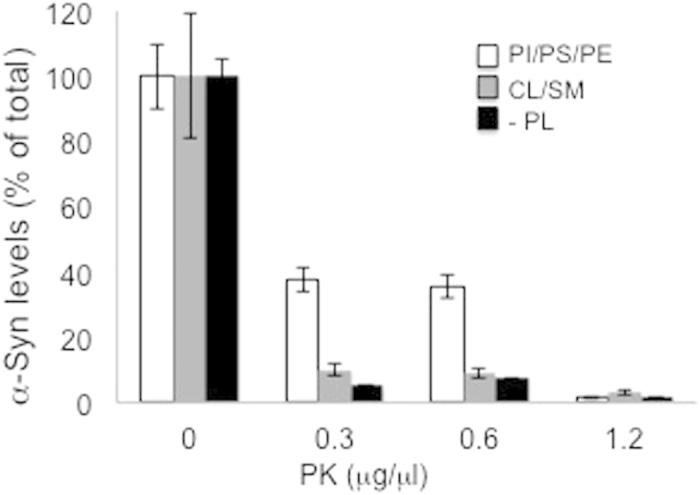
α-Syn resistance to proteinase K is acquired in the presence of phospholipids. Samples of SPRBC (5 μg protein) pre-incubated with PI/PS/PE (1:1:1) or cholesterol/sphingomyelin (1:1), at a final amount of 100 μg, or without any lipids prior to incubation with proteinase K. Proteinase-K resistant **α-**Syn levels determined by phospholipid-ELISA and presented as percent of total α-Syn, detected without Proteinase K. Mean ± SD of n = 3.

Resistance to proteinase K digestion was observed in samples of lysed PRBCs from HC and PD groups (Fig. 4a–f). Yet, no evidence for truncated **α-**Syn were detected on the Western blots (Fig. 4a,b), moreover, this resistance was lost following heat denaturation in the S-PRBC sample. To understand **α-**Syn resistance to proteinase K digestion we incubated S-PRBC samples (5 μg protein) in the presence of a mixture of phospholipids consisting of PI-PS-PE or with cholesterol/sphingomyelin (at a final amount of 100 μg), or without any lipids in the test tube, for 18 hours at 4 °C. Importantly, in a recent study we have shown that **α-**Syn specifically binds PI-PS-PE but does not bind cholesterol or sphingomyelin^19^. Following incubation with the specified lipids, samples were subjected to proteinase K digestion (0–1.2 μg/ μl) and analyzed by phospholipid-ELISA. The degree of proteinase K-resistant **α-**Syn was determined as a percent of an equivalent sample treated and handled in parallel but without proteinase K (representing total **α-**Syn). The result suggested that in the presence of PI-PS-PE, ~ 39–35% of total **α-**Syn in the test tube is resistant to digestion by proteinase K at 0.3 and 0.6 μg/ μl (Fig. 5). No resistance was obtained with cholesterol/sphingomyelin or in the absence of phospholipids. We therefore concluded that **α-**Syn associations with lipids might protect it from digestion by proteinase K.

### Higher levels of total and proteinase K resistant-α-Syn are detected by the phospholipid-ELISA than by a standard sandwich ELISA

**Figure 6:**
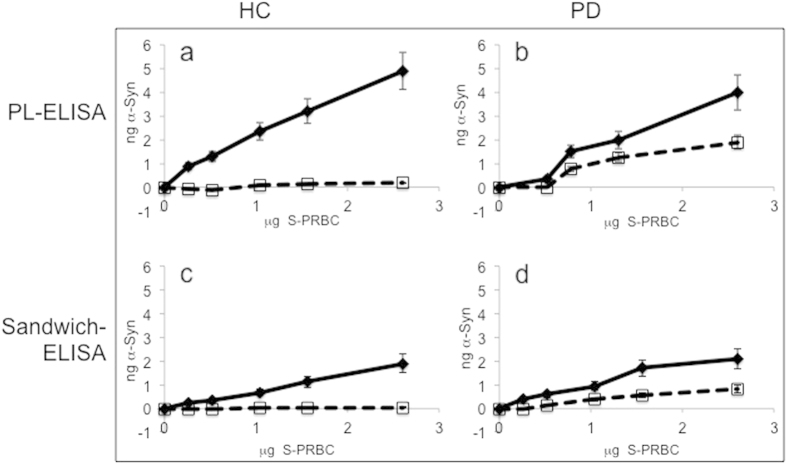
Total and proteinase K-resistant α-Syn are detected in samples of SPRBC by a standard sandwich-ELISA but with a lower efficacy. Samples of human S-PRBC (0–2.5 μg protein) were applied to a 96-well microtiter dishes and **α-**Syn levels were determined by phospholipid (PL)-ELISA (as in Fig. 2a) or a sandwich- ELISA, using anti **α-**Syn ab, Syn**-**1 (Transduction laboratories) as a capturing antibody. α-Syn detection was obtained with α-Syn #3 antibody (PL-ELISA, a and c) or C-20 antibody (Santa Cruz) (sandwich ELISA, b and d). Graph illustrates the means ± SD (n = 3 replicates) of total α-Syn (filled line) and proteinase K-resistant α-Syn (dashed line) levels of a representative sample in each group.

We sought to compare the efficacy of **α-**Syn detection by the two methods. To this aim, we measured total and proteinase K-resistant **α-**Syn levels, in samples of S-PRBC, by a reported sandwich-ELISA and in parallel, by the phospholipid-ELISA method. For sandwich-ELISA, we used a previously described protocol^20^. Consisting of a monoclonal anti **α-**Syn ab, Syn-1 (Transduction laboratories) for **α-**Syn capture and a polyclonal C-20 ab (Santa Cruz) for **α-**Syn detection. Samples of S-PRBC from healthy controls and PD patients (n = 3) were tested in parallel by the two methods. α-Syn levels were determined according to a standard curve with known amounts of purified-recombinant α-Syn (0.006–1.2 ng/μl), performed in parallel (on the same dish) with the S-PRBC samples. The calculated total **α-**Syn level (in ng) per μg protein detected for these samples by the sandwich ELISA was set at 1.44 ± 0.61. This amount is ~3–4 folds lower than total **α-**Syn level detected by the phospholipid ELISA (5.96 ± 1.05 ng/μg protein). Importantly, a similar ratio of total to proteinase K-resistant **α-**Syn levels was detected by both methods (Fig. 6).

### Lower ratio of total-to-proteinase K-resistant α-Syn in the PD group

**Figure 7:**
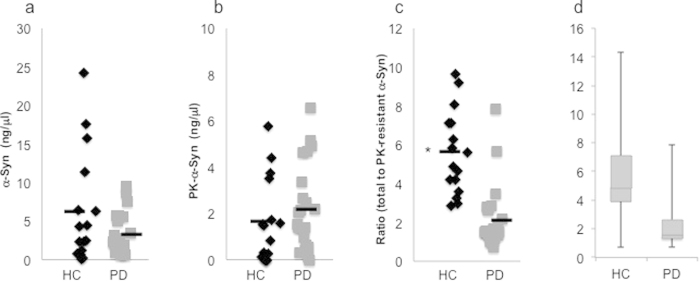
α-Syn levels in healthy controls and PD groups (**a**) Total **α-**Syn levels determined by phospholipid ELISA in healthy control (HC; n = 15); and PD (n = 21). (**b**) Proteinase K- resistant α-Syn determined by phospholipid ELISA. (**c**) The ratio of total-to-proteinase K-resistant α-Syn. Bar, represents the mean value of the group. *, P < 0.001, ttest. (**d**) The ratio of total-to-proteinase K-resistant α-Syn as in (**c**), presented in a whiskers plot. The horizontal bold lines indicate the medians; the lower part of boxes indicate the first quartile and the upper part the third quartile; the vertical lines indicate the range of values.

To find out whether RBC-expressed **α-**Syn associates with PD, we determine total and proteinase K-resistant **α-**Syn levels, in S-PRBC from 15 healthy control and 21 PD patients by phospholipid-ELISA (Fig. 7). The amount of total α-Syn in the control group was 5.47 ± 3.7 ng/μg (mean ± SD). This value was higher than the measured α-Syn signal for the PD group: 3.09 ± 1.65 ng/μg (mean ± SD). Proteinase K-resistant α-Syn levels were significantly lower in healthy controls (1.64 ± 1.46 ng/μg) than in PD patients (2.0 ± 1.2 ng/μg) mean ± SD, P < 0.001 ttest.

The ratio of total-to-proteinase K-resistant α-Syn in each group was significantly lower for the PD (1.69 ± 0.612) than for healthy controls 6.23 ± 4.09 (mean ± SD, p < 0.001, ttest; Fig. 7c). A whisker plot presenting the ratio of total-to proteinase K **α-**Syn levels (Fig. 7d) indicated that the groups differed in values of the upper and lower quartiles, while the whiskers, extending vertically from the boxes, indicated a high variability within each group and overlapping values between the two groups.

The PD group consisted of patients with implanted deep brain stimulation ((DBS), n = 12 out of 21 PD patients in the study). No effects for DBS implantation on total **α-**Syn levels (3.28 ± 3.09 ng/μg; Fig. 7a); proteinase K-resistant **α-**Syn levels (2.29 ± 2.02 ng/μg; mean ± SD; Fig. 7b); or the ratio of total-to proteinase K **α-**Syn (2.55 ± 2.149) were detected between PD patients without or with DBS implantation.

## Discussion

In this study we investigate RBC’s-expressed **α-**Syn and the potential association with PD. The rational being that peripheral pathogenic mechanisms taking place at the gastro-intestinal tract of PD patients may enhance changes in conformation or post translational modifications of RBCs-expressed **α-**Syn. The group of PD patients consists of a variable degree of disease severity and subjected to different treatment protocols. We determine total and proteinase K-resistant **α-**Syn levels by their ability to bind phospholipids. We report a significantly lower ratio of total-to-proteinase K-resistant **α-**Syn in the PD patients than in the control group, but with a certain degree of overlapping values. Our results suggest that **α-**Syn residing in RBCs associate with PD. Yet, additional markers and larger cohorts are needed to improve its reliability as a biomarker for the disease.

Reliable biomarkers are needed for early and accurate diagnosis of PD, to measure disease progression and response to therapy. A favorable candidate to serve as a biomarker is **α-**Syn, primarily due to the strong associations between **α-**Syn pathogenesis and the progression of PD. Many studies have quantified CSF levels of **α-**Syn. The majority of these studies observed lower levels of **α-**Syn in PD^21^. Importantly, levels of **α-**Syn in CSF were shown to correlate with parameters of PD^22^. In contrast, total **α-**Syn levels in plasma have been inconsistent^23^. An alternative approach has focused on measurements of specific **α-**Syn forms, which may better represent the pathogenic process, including **α-**Syn oligomers^24^ and phosphorylated **α-**Syn^23^. Nevertheless, the attempts to determine CNS-originated **α-**Syn in CSF and plasma face a major difficulty. That is, the risk of sample contamination with RBC-expressed **α-**Syn, which appears to associate with the pathogenic process of PD. Using biochemical methods, we were able to distinguish between specific **α-**Syn and non specific signals, resulting from the occurrence of peroxidases in RBCs. These peroxidases are enriched in RBC and directly react with the enhanced chemiluminescence (ECL) detection reactions. Alternative protein detection systems may potentially overcome the need for semi purification of **α-**Syn from RBCs prior to its analysis by Western blotting and ELISA.

To the best of our knowledge, our results suggest, for the first time, that RBC- originated **α-**Syn associates with PD. Larger cohorts of healthy controls and patients with PD are needed to determine whether RBC’s-**α-**Syn associates with specific parameters of PD or with PD progression. A key question is, Does **α-**Syn expressed in RBCs may associate with peripheral mechanisms of PD and provide a marker for early, pre-symptomatic diagnosis? While our current understanding does not provide an answer to this question, it is now clear that we must not neglect RBCs expressed **α-**Syn when searching for biomarkers for PD.

The occurrence of a peripheral pathogenic mechanism that precedes the CNS disease in PD was suggested by Braak^4^. Recent studies have successfully shown accumulation of **α-**Syn pathology in peripheral autonomic myenteric neurons^5^,^6^ of patients with PD. Studies in animal models provide evidence supporting a spread of **α-**Syn from the peripheral to the central nervous system^7^,^8^,^9^. Together, a growing evidence supports the Braak hypothesis claiming that PD originates as a peripheral disease and only later on spreads to the CNS.

**α-**Syn levels were determined utilizing a phospholipid-ELISA method that we developed recently^19^. The method consists of **α-**Syn capture from the sample by a mixture of phospholipids. The phospholipid mixture consists of PI and PS, which are negative phospholipids, and PE, a zwitterionic phospholipid. The preference of α-Syn to bind negatively charged phospholipids, such as PS and PI, was shown previously^25^,^26^. In addition, we found that adding PE to the mixture of phospholipids improves α-Syn’s capture from solution by the phospholipid ELISA. Importantly, early studies indicated that the natively unfolded α-Syn protein acquired its structure upon interaction with membrane phospholipids^26^. It is therefore plausible that this structure acquisition improves the detection by specific anti **α-**Syn antibodies. The results presented in Fig. 6 suggest that higher levels of total and proteinase K-resistant **α-**Syn are detectable by the phospholipid ELISA than by the sandwich ELISA. Implying that the phospholipid-ELISA method is more sensitive for **α-**Syn detection. Nevertheless, the reliability of the comparison between the two ELISA methods will benefit replication of results by other research groups that have no competing interests in one method or another.

The results suggest that proteinase K-resistant **α-**Syn form(s) occur in human RBCs. The proteinase K-resistant **α-**Syn is detectable by phospholipid binding as well as by specific anti **α-**Syn antibodies. Further purification and analyses are needed to determine the biochemical and biophysical properties of **α-**Syn protein enabling resistance to proteinase K- digestion and the relevance to the pathophysiology of **α-**Syn in RBCs. It is well accepted that amyloid structures in **α-**Syn fibrils or pathologic depositions of **α-**Syn are highly resistant to proteinase K degradation^27^,^28^. Moreover, proteinase K digestion is currently used as a research tool for pathogenic mechanisms in neurodegeneration. Yet, the short life cycle of RBCs is not expected to allow accumulation of aggregated or pathologic depositions of **α-**Syn. While the results herein demonstrate a tendency towards higher levels of proteinase K-resistant **α-**Syn in PD samples, it is important to substantiate this primary observation in a larger cohort of patients with PD, preferentially at more distinctive stages of the disease.

We show evidence suggesting that **α-**Syn resistance to proteinase K digestion may result from its associations with phospholipids. Specifically, we took advantage of **α-**Syn’s preference for binding PI-PS-PE and opposition to bind cholesterol or sphingomyelin to show that resistance to proteinase K digestion is obtained upon associates with membrane lipids. The observation may indicate an acquisition of specific structures that resist proteinase K digestion, alternatively, that the physical association of **α-**Syn with lipids protects it from proteinase K digestion.

α-Syn is mainly detected as a cytosolic protein and only a portion of it is recovered bound to membranes^29^,^30^. A recent study showed that *in vivo*, membrane-bound **α-**Syn is localized to the presynaptic terminal, while in the soma, **α-**Syn is predominantly cytosolic^31^. A relevant question that may rise from our study is, of total **α-**Syn in the sample, what portion of **α-**Syn is detected by phospholipid binding. To answer this question one may rely on alternative quantitative assays designed to quantify **α-**Syn using specific antibodies. However, the efficacy of these sandwich ELISA assays to determine total **α-**Syn may be limited by specific epitope(s) recognition of the capturing antibody. Therefore, it will be difficult to obtain a reliable answer to this question with the currently available research tools.

The group of patients recruited to this study represents a heterogeneous group. Including, a variable degree of disease severity; different duration of disease; and different protocols of drug administration, with or without implantation of deep brain stimulation (DBS). This study therefore may highlight the high potential residing in PRBC-originated **α-**Syn but may not allow conclusive results. It is important to include drug naïve patients and long-term follow-up, in future studies, to be able to determine the reliability of PRBC-originated **α-**Syn as a biomarker reflecting the prognosis, diagnosis and progression of the disease.

In conclusion, our data suggest that RBCs-expressed **α-**Syn may potentially serve as an indicator for PD. Specifically, that the ratio of total-to-proteinase K-resistant **α-**Syn, detected in human PRBCs, associate with pathogenic mechanisms involved in PD. In addition, we show that the biochemical property of **α-**Syn that enables it to bind phospholipids, provides a promising tool for diagnosis.

## Methods

### Participants

This study was performed in accordance with the Declaration of Helsinki and approved by the Hadassah Hospital institutional review board for experiments with human subjects. Signed consent was obtained from all participants before their inclusion in the study. The diagnosis of PD was made according to the Hoehn and Yahr scale at the Department of Neurology at Hadassah Hospital.

15 healthy individuals, mean age 55.6 ± 17.9 years and 21 individuals with PD, mean age 64.1 ± 6.9 years (Table 1) were recruited to the study. The PD group consisted of individuals that were clinically diagnosed and routinely examined. The group consists of individuals with a variable degree of disease severity, represented by the unified Parkinson’s disease rating scale–motor (UPDRS) score of 3–60 points and Hoehn and Yahr score of 2.6 ± 0.9 (mean ± SD). Disease duration is 10.1+/− 6.5 years. Patients are treated either with dopamine precursor, dopamine agonist or a MAO-B inhibitor, or a combination of these drugs. Twelve out of the 21 individuals with PD subjected to this study were implanted with a deep brain stimulation (DBS) system.

Control subjects were healthy individuals with no apparent neurological or known psychiatric symptoms and no signs of dementia. The control subjects were spouses of or otherwise non-genetically related to the PD patients.

### Blood collection, separation and analysis

Blood samples were collected in EDTA tubes and processed within 2–3 hours of collection.

Three to five milliliters of blood were spun at 1000 × g for 10 minutes at 4 °C. The cell pellet was separated from the plasma and washed twice in phosphate-buffered saline at the original volume. The upper layer of cells was discarded to remove white blood cells. Packed red blood cells (PRBCs) were lysed by re-suspending the pellet 1:10 v/v in DDW. The lysed sample was spun at 20,000 × g to remove cells and debris. The supernatant and plasma were stored in aliquots at −80 °C.

For analysis, samples were thawed and diluted 1:5 (lysed PRBC) or 1:2 (plasma) in DDW. Samples of PRBCs were treated with proteinase K (at 0.6 μg/μl) at 37 °C for 30 minutes or left untreated. Samples were then heat denatured at 100 °C (dry block) for 10 minutes, followed by a spin down at 20,000 × g for 30 minutes prior to analysis of **α-**Syn by phospholipid ELISA. The resulting supernatant (S-PRBC) was transferred to clean tubes. Samples were applied onto the phospholipid-ELISA microtiter plate at 0–5 μg protein per well. Each S-PRBC sample was analyzed in triplicate on two separate dates. Samples of plasma were similarly heat-treated and centrifuged to produce the S-plasma.

### Human brains

Frozen aliquots of proteins extracted from human control brains (frontal cortex), obtained and prepared as previously described^32^ were analyzed. Human brain samples (n = 3) consisted of the soluble fraction of high-speed cytosols (post-370,000 × g)^29^.

### Phospholipid ELISA assay^19^

A PolySorp, 96-well ELISA plate (Thermo Scientific) was coated with a mixture of phospholipids dissolved in methanol in a final amount of 100 μg/well and incubated overnight at 4 °C for complete evaporation of methanol. Blocking was performed with 100 μl/well of 1% BSA (fatty acid-free) in PBS for one hour at 37 °C, followed by one wash with PBS. Samples consisting of the soluble fraction of PRBC (S-PRBC), plasma (S-plasma) or human brains, were added in triplicate at a final volume of 100 μl/well. Plates were incubated for 3 hours at 37 °C. Following incubation, samples were removed and the wells were washed 4 times with PBS. A mouse anti-human a-Syn antibody (α-Syn#10) was diluted 1:10,000 in 1% BSA in PBS. Following incubation for one hour at 37 °C, the wells were washed 3 times and incubated for one hour at 37 °C with a horseradish-peroxidase-conjugated donkey anti-mouse secondary antibody at 1:8000 (Jackson Laboratories). Following 3 washes with PBS, 50 μl of TMB One Component Microwell Substrate (SouthernBiotech, Birmingham, Alabama, USA) were added per well. The reaction was terminated with 50 μl/well of 1M H_2_SO_4_. Absorbance at 450 nm was determined using a plate reader (EL808 Ultra Microplate Reader, Bio-Tek Instruments, VT, USA). The amount of α-Syn was determined for each plate according to a standard curve using recombinant α-Syn (at 0–50 ng/well) performed in parallel to the tested samples.

To assess whether proteinase K-resistant α-Syn is detectable by phospholipid ELISA, PRBC samples (20 μg protein) were treated with increasing concentrations of proteinase K (0, 0.3, 0.6, 1.2 μg/μl) for 30 minutes at 37 °C. Following the treatment, the samples were either left untreated (samples reacted with anti β-actin antibody) or heated at 100 °C for 10 minutes and spun at 20,000  × g to obtain the S-PRBC (samples reacted with anti **α-**Syn antibody). Samples were then subjected to analysis by phospholipid ELISA and Western blotting.

### Sandwich ELISA

Total and proteinase K-resistant α-Syn levels were determined in samples consisting of the soluble fraction of PRBC (S-PRBC, 0–2.5 μg protein), using an in-house ELISA assay, as recently described^20^. α-Syn levels were determined according to a standard curve with known amounts of purified-recombinant α-Syn (0.006–1.2 ng/μl), performed in parallel to the tested sample.

### Western blotting

Protein samples were loaded onto 10% SDS-polyacrylamide gels. Following electrophoresis, proteins were transferred to an immunobilon-P membrane (Millipore, Bedford, MA, USA).

Membranes were blocked for 1 hour with 5% nonfat milk in Tris-buffered saline containing 0.05% Tween 20 (TBST). Membranes were probed with the specified primary antibodies diluted in 5% nonfat milk in TBST. Anti **α-**Syn ab: **α-**Syn#10^19^, a human **α-**Syn specific; and anti **α-**Syn#3, recognizes mouse and human **α-**Syn, anti β-actin ab (Sigma-Aldrich). After washes with TBST, the blots were incubated with secondary antibodies conjugated to horseradish-peroxidase. Immunoreactive bands were detected by the enhanced chemiluminescence reagent (ECL) (Pierce, CT, USA).

## Additional Information

**How to cite this article**: Abd-Elhadi, S. *et al.* Total and Proteinase K-Resistant a-Synuclein Levels in Erythrocytes, Determined by their Ability to Bind Phospholipids, Associate with Parkinson’s Disease. *Sci. Rep.* **5**, 11120; doi: 10.1038/srep11120 (2015).

## Figures and Tables

**Table 1:** Demographic and clinical information.

	**HC**	**PD**
Age (years), mean (SD)	55.6 (17.9)	64.0 (6.9)
Female / male (total), No.	6/9 (15)	7/14 (21)
Age at diagnosis, mean (SD)	na	53.8 (8.2)
Disease duration, (years)	na	10.1 (6.5)
DBS, No.	0	12
Hoehn and Yahr grade, mean (SD)	na	2.6 (0.9)
